# Exploration of potential novel drug targets for rheumatoid arthritis by plasma proteome screening

**DOI:** 10.1371/journal.pcbi.1013333

**Published:** 2025-09-25

**Authors:** Zhiqiang Ma, Ran Chen, Zibo Feng

**Affiliations:** 1 Department of Cardiovascular Medicine, The 2nd Affiliated Hospital, Jiangxi Medical College, Nanchang University, Nanchang, Jiangxi, China; 2 Department of Cardiology and Hypertension, Yanbian University Hospital, Yanji, Jilin, China; 3 The Laboratory of Metabolic Disorders and Vascular Aging, Liyuan Hospital, Tongji Medical College, Huazhong University of Science and Technology, Wuhan, Hubei, China; 4 Department of Wound Repair Surgery, Liyuan Hospital, Tongji Medical College, Huazhong University of Science and Technology, Wuhan, Hubei, China; KU: The University of Kansas, UNITED STATES OF AMERICA

## Abstract

**Background:**

Circulating proteins play a critical role in rheumatoid arthritis (RA), yet few have been targeted therapeutically. This study aimed to identify novel protein targets for RA therapy.

**Methods:**

We conducted a comprehensive proteome-wide Mendelian Randomization (MR), colocalization analysis, and summary-data-based MR (SMR) to explore potential causal relationships between plasma proteins and RA, with an overall sample size of 1,148,608. The GWAS data on plasma proteins were obtained from the FinnGen study, the UK Biobank Pharma Proteomics Project and Iceland GWAS data. Then, validation of key molecules’ differential expression pattern was done using external transcriptomic data from RA patients, while the Drug Signatures Database (DsigDB) was used to identify potential therapeutic drugs. Drugs and target proteins interactions was evaluated with molecular docking and molecular dynamics simulations approaches. Potential side effects of plasma proteins associated with RA were elucidated by phenome-wide association study (Phe-WAS) approach.

**Results:**

Genetically predicted levels of 68 plasma proteins were associated with RA. After colocalization and SMR analysis, 6 plasma proteins (FCRL3, SUGP1, TNFAIP3, EHBP1, HAPLN4, and CILP2) have been passed all tests and identified as having potential as therapeutic targets for RA. Further Receiver operating characteristic curve (ROC) analysis indicated that three protiens (CILP2, TNFAIP3 and EHBP) have a good potential as biomarkers for RA. Differential gene analysis showed the downregulation of HAPLN4, FCRL3, EHBP1 and TNFAIP3 in RA, as well as the upregulation of CILP2 in RA. Further Phe-WAS suggested that targeting these proteins may have potential side effects.

**Conclusion:**

Our study investigated the causal relationships between plasma proteins and RA, deepening our understanding of the molecular mechanisms and facilitating the development of new therapeutic drugs.

## Author summary

Rheumatoid arthritis (RA) is an autoimmune disease characterized by chronic inflammation of the synovial joints, with a global prevalence estimated at approximately 0.2 - 1%. As patients age, RA can lead to joint stiffness, destruction, deformities, disability and other complications, resulting in significant social and economic burdens. Current treatment options, such as non-steroidal anti-inflammatory drugs and biologic disease-modifying antirheumatic drugs (DMARDs), while improving patient prognosis, still face significant limitations including variable patient responses, drug resistance, and adverse effects, which hinder effective management of the disease. Therefore, the identification of new biomarkers to enhance early diagnosis and treatment is critical. Based on a large-scale cohort, this study integrated Mendelian randomization analysis with bioinformatics approaches to systematically evaluate the potential causal relationships between plasma proteins and RA. Several novel proteins were identified to be significantly associated with RA, offering new biological insights into its pathogenesis. These proteins may also serve as promising therapeutic targets, providing a theoretical foundation for the development of more effective treatment strategies. Overall, this study advances the understanding of RA pathophysiology and offers new directions for clinical diagnosis and intervention, addressing a critical need in the field.

## Introduction

Rheumatoid arthritis (RA) is an autoimmune disease characterized by chronic inflammation of the synovial joints, often leading to joint destruction and functional loss. The joint damage caused by RA differs from that resulting from other diseases. For instance, Hashimoto’s hypothyroidism primarily affects thyroid function, leading to metabolic disturbances with minimal direct joint damage. In contrast, primary metabolic gout is caused by the accumulation of uric acid crystals, resulting in acute joint inflammation rather than the chronic inflammation associated with RA. Currently, the prevalence of RA is estimated at a range of 0.2-1%, with female having a 2–3 times higher incidence than males [1,2]. RA typically manifests in middle age, and as the disease progresses, patients gradually develop joint stiffness, destruction, deformities, and disability. Indeed, RA patients frequently suffer from multiple organ complications, such as stroke, myocardial infarction, and interstitial lung disease, which significantly increase their mortality and hospitalization rates [3]. These multisystem damages, including those affecting the cardiovascular system, respiratory system, and musculoskeletal system, not only exacerbate patient suffering but also impose a heavy burden on society and healthcare resources.

RA progression from preclinical stage to a chronic condition involves multiple pathogenic pathways and cellular lineages, varying across patients and complicating treatment. RA current therapeutic pattern primarily include symptomatic treatments with non-steroidal anti-inflammatory drugs and glucocorticoids, as well as disease-modifying antirheumatic drugs (DMARDs) [4]. Among these, biological DMARDs specifically target plasma proteins, directly intervening in the core pathological mechanisms of RA. Numerous studies have demonstrated that biological DMARDs significantly improve patient prognosis, underscoring the effectiveness and potential of plasma protein-targeted therapies [5,6]. In recent years, biological DMARDs have increasingly become the first-line treatment for RA patients with comorbidities, further establishing their importance in clinical practice [7]. However, despite these therapeutic advances, significant limitations persist, including variable patient responses, potential drug resistance, and severe side effects such as increased infection risk and organ toxicity [8]. The pathogenesis of RA is highly complex, involving a wide array of molecules and signaling pathways. Current methods for target screening have proven inefficient in addressing the multitude of potential targets. Moreover, randomized controlled trials face substantial limitations in investigating the causal relationships between thousands of proteins and RA, particularly in the context of complex, multifactorial disease mechanisms. Randomized controlled trial (RCTs) often fail to fully eliminate confounding factors, thereby limiting the external validity of their findings. These challenges significantly hinder the efficiency of identifying and validating novel therapeutic targets. Consequently, more efficient research methodologies are urgently needed to accelerate the discovery and validation of new drug targets, thereby advancing RA treatment.

Mendelian randomization (MR) is an epidemiological technique that leverages genetic variants reliably associated with potentially modifiable risk factors to determine their causal effects on disease risk [9,10]. Because genetic variants are randomly assigned at conception, they are largely independent of confounding factors, thereby reducing the risk of confounding. Genome-wide association studies (GWAS) have identified specific single nucleotide polymorphisms (SNPs) that regulate protein expression. These SNPs correspond to quantitative traits of protein abundance, commonly referred to as protein quantitative trait loci (p-QTL). By using these p-QTL as instrumental variables (IVs), it is possible not only to enhance the reliability of target discovery but also to gain new insights into biological mechanisms, which could contribute to the development of more effective therapeutic strategies.

Our study was a comprehensive protein-wide MR analysis, investigating the causal relationship between plasma proteins and RA. Colocalization and summary-data-based MR (SMR) analyses were employed to filter these proteins. Depth apprehension of proteins functions and results reliability validation was achieved by employing enrichment, protein-protein interaction (PPI), differential expression analyses and receiver operating characteristic curve (ROC) on transcriptomic data. Moreover, we used the Drug Signatures database (DsigDB) to predict upstream drugs targeting key proteins, and molecular docking, while molecular dynamics simulations were applied to assess the binding activity and stability between the drugs and target proteins. Finally, phenome-wide association studies (Phe-WAS) were conducted to assess the potential beneficial or adverse effects of the most promising protein on other phenotypes.

## Methods

### Study design and overview

**Fig 1:**
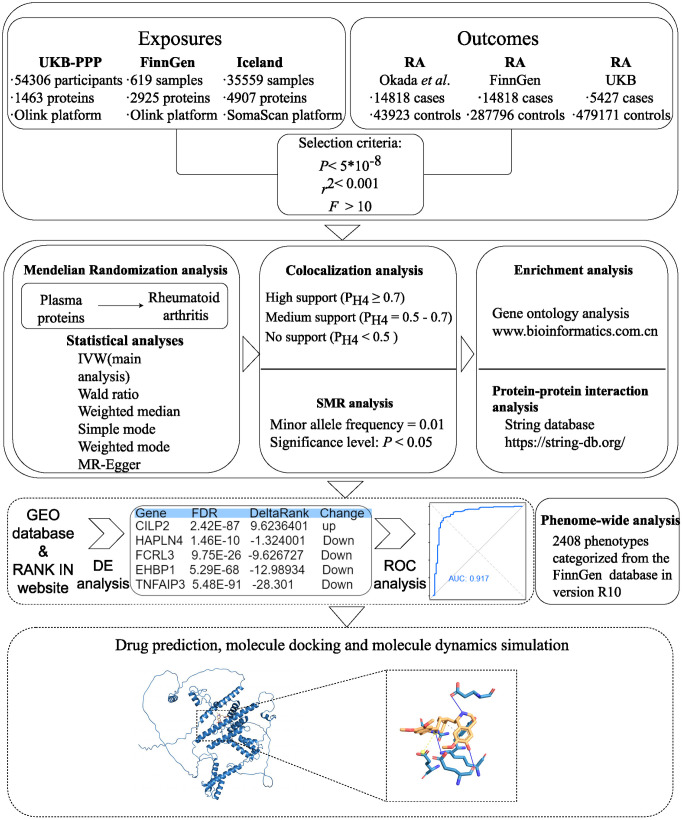
Overview of the study design in this study. RA, rheumatoid arthritis; UKB, UK Biobank; IVW, inverse‐variance weighted; SMR, summary-data-based mendelian randomization; DE, differential expression; ROC, receiver operating characteristic; AUC, area under the curve.

This MR study adheres to the Reporting of Observational Studies in Epidemiology Using Mendelian Randomization STROBE Guidelines [11]. A schematic overview of the study design and data sources is detailed in Fig 1. Briefly, we designed the study as follow: 1) For the exposure, we extracted plasma protein p-QTL data from UK Biobank (UKB), FinnGen, and Iceland data as proxies. For the outcomes, we retrieved GWAS data on RA from both the study by Okada *et al.*, UKB and FinnGen; 2) Protein-wide MR analysis was applied in order to avoid sample and sampling overlap; 3) Colocalization analysis and SMR analysis were conducted to identify proteins with potential drug targets; 4) Enrichment analysis and PPI analysis were conducted to identify the interactions among core proteins, the biological processes they are involved in, and their associations with disease; 5) Differential expression and ROC analyses were utilized to validate potential core proteins as biomarkers for RA; 6) Predict potential upstream targeted drugs for the core proteins and validate the binding stability between the drugs and proteins through molecular docking and molecular dynamics simulations; 7) To evaluate the potential beneficial or adverse effects of plasma proteins on other phenotypes, we conducted a Phe-WAS.

### MR assumptions

In our study, we adopted and implemented the following three key assumptions of MR: 1) genetic instruments are significantly associated with exposure of interest; 2) genetic instruments are not related to any confounding factors of the exposure-outcome association; 3) genetic instruments affect the outcome only via the exposure.

### GWAS data sources

The GWAS data on plasma proteins were obtained from the FinnGen study, the UK Biobank Pharma Proteomics Project (UKB-PPP) (https://www.synapse.org/#!Synapse:syn51365301) and Iceland GWAS data. The FinnGen study is a large-scale genomics initiative that has analyzed over 500,000 Finnish biobank samples and correlated genetic variation with health data to understand disease mechanisms and predispositions. We used the Finnish proteomics data from Olink, which includes 619 samples involving 2,925 plasma proteins [12]. The project is a collaboration between research organisations and biobanks within Finland and international industry partners (https://www.finngen.fi/en/for_researchers). The UKB-PPP is a precompetitive biopharmaceutical consortium characterizing the plasma proteomic profiles of 54,219 UK Biobank (UKB) participants, measuring 2,941 protein analytes and capturing 2,923 unique proteins [13]. The authors identified 14,287 significant primary associations across 3,760 independent genetic regions at a multiple testing-corrected threshold of *P* < 1.7E-11. Additionally, at a less stringent single-phenotype genome-wide significance threshold of *P* < 5E-08, the authors found 14,731 additional associations across 2,519 proteins. For quality control, the authors analyzed a total of 171,377,949 data points. Of these, 3.3% had QC/assay warnings, 0.7% were identified as outliers, and 2.6% were flagged for potential sample swapping. In the UKB-PPP, the mean age of the included subjects was 56.7 years, and 45% of the enrolled participants were male patients. The mean BMI for all subjects was 27.4 kg/m^2^, and 46% of the subjects had a previous history of smoking. The Iceland GWAS data are derived from two main projects: the Icelandic Cancer Project (52% of participants) and various genetic programs at deCODE Genetics Project (48% of participants). The study, based on the SomaScan platform, identified 28,191 genetic associations of 4,907 aptamers in a cohort of 35,559 Icelandic individuals [14]. The authors restricted the search for conditional variants to the set of variants with marginal (unconditional) *P* values less than 5E-06. For quality control, the authors calculated the correlation of log-transformed relative fluorescence units across all 5,284 aptamers for each sample pair, yielding a high median correlation of 0.94. Samples with a correlation below 0.82 were excluded. Aptamers for non-human proteins and aptamers listed as deprecated by SomaScan as well as aptamers mapping to multiple genes were excluded, leaving 4,907 aptamers that were included in the pQTL analysis.

For outcome, summary-level data on RA were obtained from the study from Okada *et al.* which includes 58,284 individuals (14,818 cases and 43,923 controls), the FinnGen database, which includes 302,614 individuals (14,818 cases and 287,796 controls), the study from the BioBank Japan (BBJ) database, which includes 212,453 individuals (4,199 cases and 208,254 controls) and the UKB database, which includes 484,598 individuals (5,427 cases and 479,171 controls) [12,15,16]. For all data sources, see S1 Table. This study is a Mendelian randomization analysis, and all GWAS data used have been approved by the respective original research ethics committees. No additional ethical approvals were required, as our study utilized publicly available summary-level data.

### Genetic instruments selection

The steps for selecting optimal genetic instruments were as follows: 1) The SNP within a vicinity of ± 1 Mb around the gene region (cis-pQTL) served as instrumental variables; 2) We selected SNPs with significant plasma protein associations (P < 5E-08) based on the criteria mentioned in previous studies; 3) The linkage disequilibrium of IVs were removed to ensure mutual independence of these IV (*r*^2^ = 0.001, kb = 10,000); 4) To quantify the strength of IV, we calculated F-statistics, and a threshold of the F-statistics >10 was typically recommended for MR analyses.

### MR analysis

Primary analysis for the MR study was the inverse‐variance weighted (IVW) method, which provides a robust causal estimate in the absence of directional pleiotropy. When only one SNP was available for a particular protein, we applied the Wald ratio method. Supplementary analyses were conducted using the weighted median and MR-Egger methods. The weighted median method can provide consistent estimates when more than 50% of the weight comes from valid instrument variants [17]. MR-Egger regression can generate estimates after accounting for horizontal pleiotropy albeit with less precision [18]. If the IVW method result is significant (*P* < 0.05), even if the results of other methods are not significant, and no pleiotropy and heterogeneity were identified, it can be regarded as a positive result, provided that the beta values of the other methods are in the same direction. To correct for multiple comparisons for multiple hypotheses, a false discovery rate (FDR) adjusted *P*-value was used in the main IVW analyses (*P* < 0.05 was judged significant based on the criteria mentioned in previous study) [19]. After FDR adjustment, the results that met the threshold were considered as significant causal associations. Then, we performed tests for directional horizontal pleiotropy by MR-Egger intercept (**P* *< 0.05 was judged significant). The study utilized R 4.2.2 software and the R packages “TwosampleMR” for analysis.

### Colocalization analysis

The method was referenced to the previous study [20]. To confirm whether identified associations of proteins with RA were driven by linkage disequilibrium, colocalization analysis was performed. The colocalization analysis was based on a Bayesian model that assesses the support for five exclusive hypotheses: 1) No association with either trait; 2) Association with trait 1 only; 3) Association with trait 2 only; 4) Both traits are associated, but distinct causal variants for the two traits; and 5) Both traits are associated, and the same shared causal variant for both traits. A posterior probability is provided for each hypothesis testing (H0, H1, H2, H3, and H4). We set prior probabilities of the SNP being associated with trait 1 only (p1) at 1 × 10 ⁻ ⁴; the probability of the SNP being associated with trait 2 only (p2) at 1 × 10 ⁻ ⁴; and the probability of the SNP being associated with both traits (p12) at 1 × 10 ⁻ ⁵. If the posterior probability for shared causal variants (P_H4_) was ≥ 0.8, it was considered to have colocalization support; otherwise, it was considered unsupported. The analysis was performed using the coloc package in R software (4.2.2).

### SMR analysis

The SMR method can be interpreted as a technique to assess whether the effect size of a SNP on a phenotype is mediated by gene expression. This approach is therefore useful for prioritizing genes underlying GWAS hits for subsequent functional studies. In our study, we employed SMR analysis to evaluate the relationship between alterations in target gene expression and the risk of RA. Initially, we conducted SMR analysis using cis-eQTL data from the eQTL Gene Consortium (https://www.eqtlgen.org/phase1.html), which provided a substantial sample size (n = 31,684) to identify SNPs associated with the expression of genes targeted by the corresponding plasma proteins. For replicate analysis, we acquired tissue-specific cis-eQTLs from 49 tissues (n = 15,201) in the GTEx v8 (https://yanglab.westlake.edu.cn/data/SMR/GTEx_V8_cis_eqtl_summary.html) project to investigate the tissue-specific associations and potential target effects of drug-targeted genes. All analysis were performed using SMR software, version1.03 (https://yanglab.westlake.edu.cn/software/smr/#Overview). A significance level of *P* < 0.05 was set for the SMR analysis.

### Enrichment analysis and PPI analysis

Perform gene ontology (GO) enrichment analysis, Kyoto Encyclopedia of Genes and Genomes (KEGG) pathway enrichment analysis, and data visualization using the “GO_KEGG” enrichment analysis module on the Bioinformatics website [21] (https://www.bioinformatics.com.cn). The Bioinformatics website is an online platform for data analysis and visualization. PPI analysis was performed through STRING [22] (https://string-db.org/) or geneMANIA (https://genemania.org/) with interaction score as 0.400 [23].

### Selection, merging, and differential analysis of bulk transcriptomic datasets

A total of 11 human peripheral blood (cell) or synovial tissue transcriptomic or microarray datasets related to RA were selected from the Gene Expression Omnibus (GEO) database (https://www.ncbi.nlm.nih.gov/geo/) [24–30], with detailed information provided in S2 Table. Gene IDs from the original expression matrices were mapped to gene symbols according to the annotation files specific to each analysis platform. The genes common to both the blood and synovial datasets were retained separately, and then the blood or synovial datasets were merged into a composite matrix based on matching gene symbols. To integrate gene expression profiles across platforms, we identify and correct batch effects, and calculate differential gene expression using the Rank-In algorithm (http://www.badd-cao.net/rank-in/index.html), which is an advanced genomic data integration method [31]. The composite matrix, along with sample group labels and gene expression platform labels, was formatted according to the specifications of the Rank-In website and submitted to the Rank-In web server. The server returned a normalized gene expression matrix and a list of differentially expressed genes for downstream analysis. This list included gene names, FDR values, and DeltaRank values. The FDR method was used to balance the detection of statistically significant genes against the risk of false positives. In this study, genes with FDR < 0.01 and absolute DeltaRank values > 1 were considered significant.

### ROC analysis

To evaluate the discriminative ability of individual candidate genes, each gene was treated as a continuous univariate classifier to distinguish RA from control samples. The dataset was randomly divided into a training set (70%) and a test set (30%) using a fixed seed (set.seed = 123) to ensure reproducibility. ROC curves and corresponding area under the curve (AUC) values were calculated on the test set using the pROC R package in R software (4.2.2). To address class imbalance (666 RA vs. 177 HC), random oversampling was applied to the training set using the ovun.sample() function from the ROSE package. The test set remained unaltered to ensure an unbiased evaluation under the original sample distribution. The potential of its role as a molecular biomarker was evaluated based on the value of the AUC [32]. In general, AUC values are interpreted as follows: 0.5 - 0.6 (failed), 0.6 - 0.7 (worthless), 0.7 - 0.8 (poor), 0.8 - 0.9 (good), > 0.9 (excellent) [33,34].

### Random forest classification

To evaluate the predictive performance of the candidate genes, a random forest classification model was constructed. The gene expression matrix was used as input, and sample group labels (RA vs. healthy control) were used as the output variable. The model was trained using the randomForest package in R, with default parameters and 500 decision trees. To assess the classification performance of the model, ROC curve analysis was conducted, and the AUC was calculated using the pROC package. Feature importance was evaluated based on the mean decrease in Gini index, which reflects the relative contribution of each variable to the overall classification accuracy.

### Drug prediction and molecular docking

Computer-aided virtual screening is an effective method for identifying small molecule drugs with binding affinity to target receptors. Structure-based pharmacophore strategies have been successfully used for screening small molecule lead compounds in drug development. Molecular docking and dynamic simulation are also considered practical methods for analyzing intermolecular interactions, explaining binding affinity, and stability. Therefore, combining pharmacophore models with molecular docking will achieve more effective matches [35]. Drug molecule was predicted using the DSigDB [36] via Enrichr [37] based on key molecules. Enrichr is a popular web portal with a vast array of gene-set libraries to explore gene-set enrichment across the genome. DSigDB serves as a global archive for identifying targeted drug substances associated with genes or proteins. The compound structures are obtained from the PubChem [38] database (https://pubchem.ncbi.nlm.nih.gov/), and the protein structures are obtained from the PDB [39] database (https://www.rcsb.org/) or AlphaFold database [40,41](https://alphafold.ebi.ac.uk/). All AlphaFold-derived models used in this study were sourced from AlphaFold DB version 4 and generated using the AlphaFold Monomer v2.0 algorithm. Using the online molecular docking program CB-dock2 [42] (https://cadd.labshare.cn/cb-dock2/index.php) for docking small molecular compounds with proteins, employing template-free blind docking methods. The docking results are visualized using PyMOL [43]. In molecular docking studies, binding energy is a fundamental metric for evaluating ligand-receptor interactions. Binding energies less than 0 kcal/mol indicate a spontaneous binding process, confirming the thermodynamic feasibility of the ligand-receptor interaction [44]. Specifically, a binding energy threshold of -7.2 kcal/mol is often used as a benchmark for strong molecular interactions, characterized by increased affinity and specificity for the receptor [45].

### Molecular dynamics simulation

The best conformation and binding energy of the protein-ligand complex were used for the molecular dynamics simulation analysis [46]. In the present study, the GROMACS Program package [47] and CHARMM36 force field [48] were used to simulate the molecular dynamics of the complexes in the TIP3P (Transferable Intermolecular Potential 3P) water model. Ions (Na^+^ or Cl^−^) were added to neutralize charges wherever necessary. The systems were neutralized, and energy minimized. Then, the systems were heated from 0 K to 300 K within 100 ps in NVT (Number of particles, Volume and Temperature) ensemble with normal temperature (300 K) and another 100 ps in NPT (Number of particles, Pressure and Temperature) ensemble with normal pressure (101 kPa) [49,50]. After heating and equilibration, the docked complexes were subjected to production molecular dynamics run for 100 ns after the system reached dynamic equilibrium. The simulation performed in triplicates (n = 3) and the geometric properties of the protein-ligand complexes, such as root mean square deviation (RMSD), radius of gyration (Rg), root mean square fluctuation (RMSF), and number of hydrogen bonds (H-bonds) were calculated using g_rms and g_energy programs respectively [51].

### Phe-WAS analysis

Phe-WAS is a powerful tool for evaluating associations between SNPs or phenotypes and a wide array of phenotypes spanning the entire phenome. To determine whether RA associated with plasma proteins are linked to other traits and diseases, Phe-WAS was conducted using the FinnGen database version R11, which encompasses 2,447 phenotypes. Similar to MR analysis, the IVW method was employed as the primary analysis method. A *P*-value < 0.05 was considered statistically significant. All analyses were performed using the TwoSampleMR (version 0.6.8) and MendelianRandomization (version 0.8.0) in R Software 4.4.2 (https://www.R-project.org)

## Results

### Genetic instruments

In our analysis, we extracted valid IVs from plasma proteins GWAS based on the selection criteria. Among IV included in the final analysis, all F-values are greater than 10, indicating that weak instrument bias is unlikely to be significant.

### Causal effects of plasma proteins with RA

#### Proteins from FinnGen.

**Fig 2:**
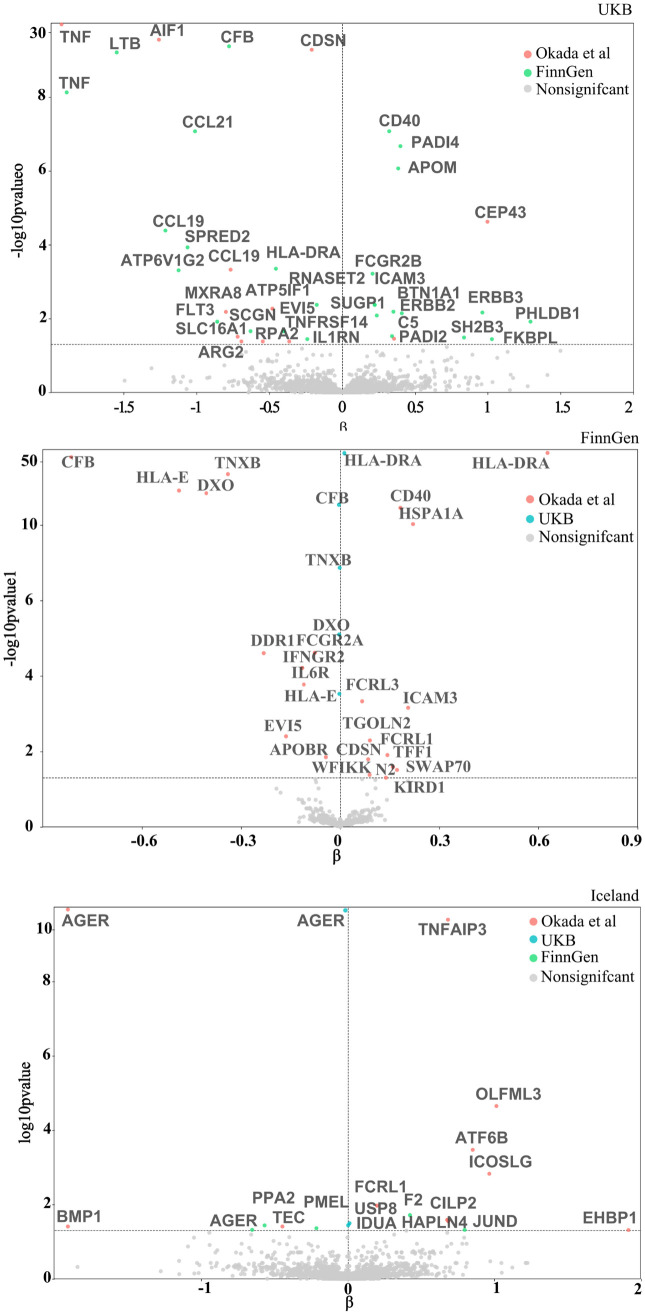
Volcano plot of MR analysis. After FDR adjustment, results of the plasma protein and RA proteome-wide MR analysis from the the FinnGen (A), the UKB (B) and the Iceland (C). Proteins on the left side of the dashed line are protective, while those on the right are risk proteins. Different colors represent the various sources of outcome GWAS data. FDR, false discovery rate; MR, Mendelian randomization; RA, rheumatoid arthritis; UKB, UK Biobank.

As shown in Fig 2A, when the RA data is sourced from the study by Okada *et al.*, a total of 429 proteins are included in the primary analysis. After FDR adjustment, 22 proteins are significantly associated with the risk of RA. Among these, negative causal associations with RA were observed for 10 proteins (APOBR, CFB, DDR1, DXO, EVI5, FCGR2A, HLA-E, IFNGR2, IL6R, and TNXB), while positive causal associations were observed for 12 proteins (CD40, CDSN, FCRL1, FCRL3, HLA-DRA, HSPA1A, ICAM3, KLRD1, SWAP70, TFF1, TGOLN2, and WFIKKN2). Per standard deviation (SD) increase in genetically predicted levels of each protein, the odds ratio (OR) of RA ranged from 0.44 [95% confidence interval (CI), 0.41 - 0.48; *P* = 7.96E-73] for CFB to 4.50 (95% CI, 4.09 - 4.95; P = 1.51E-203) for HLA-DRA.

As shown in Fig 2A, when the RA data is sourced from UKB, a total of 636 proteins are included in the replication analysis. After FDR adjustment, five proteins are significantly associated with the risk of RA. Among these, negative causal associations with RA were observed for four proteins (CFB, DXO, HLA-E, and TNXB), while a positive causal association was observed for HLA-DRA. Per standard deviation increase in genetically predicted levels of each protein, the OR of RA ranged from 0.99 (95% CI, 0.995 - 0.996; *P* = 4.86E-13) for CFB to 1.01 (95% CI, 1.01 - 1.02; *P* = 1.53E-50) for HLA-DRA. S3 Table displays the full MR results.

#### Proteins from UKB.

As shown in Fig 2B, when the RA data is sourced from the study by Okada et al., a total of 1,456 proteins are included in the primary analysis. After FDR adjustment, 27 proteins are significantly associated with the risk of RA. Among these, negative causal associations with RA were observed for 13 proteins (AIF1, ATP6V1G2, CCL19, CCL21, EVI5, FLT3, HLA-DRA, IL1RN, LTB, RNASET2, SCGN, SPRED2, and TNF), while positive causal associations were observed for 14 proteins (APOM, BTN1A1, C5, CD40, DXO, ERBB2, ERBB3, FCGR2B, FKBPL, ICAM3, PADI4, PHLDB1, SH2B3, and SUGP1). Per SD increase in genetically predicted levels of each protein, the OR of RA ranged from 0.17 (95% CI, 0.10 - 0.28; *P* = 1.65E-09) for TNF to 80.25 (95% CI, 6.74 - 955.14; *P* = 0.047) for DXO.

When the RA data is sourced from FinnGen, a total of 1,578 proteins are included in the replication analysis. After FDR adjustment, 12 proteins are significantly associated with the risk of RA. Among these, negative causal associations with RA were observed for 10 proteins (AIF1, ARG2, ATP5IF1, CCL19, CDSN, MXRA8, RPA2, SLC16A1, TNF, and TNFRSF14), while positive causal associations were observed for two proteins (CEP43 and PADI2). Per SD increase in genetically predicted levels of each protein, the OR of RA ranged from 0.16 (95% CI, 0.12 - 0.21; **P* *= 3.62E-30) for TNF to 2.71 (95% CI, 1.87 - 3.92; *P* = 2.38E-05) for CEP43. S3 Table displays the full MR results.

To investigate the causal relationship between plasma proteins and RA in East Asian populations, we used RA data from the BBJ database as the outcome. After FDR adjustment, 5 proteins are significantly associated with the risk of RA. Among these, negative causal associations with RA were observed for 4 proteins (MICB, PILRA, PILRB, and FCRL3), while a positive causal association was observed for LRP11. S3 Table displays the MR results

#### Proteins from Iceland.

As shown in Fig 2C, when the RA data is sourced from the study by Okada et *al.*, a total of 1,264 proteins are included in the primary analysis. After FDR adjustment, 11 proteins are significantly associated with the risk of RA. Among these, negative causal associations with RA were observed for three proteins (AGER, TEC, and BMP1), while positive causal associations were observed for eight proteins (TNFAIP3, OLFML3, ATF6B, ICOSLG, FCRL1, CILP2, HAPLN4, and EHBP1). Per SD increase in genetically predicted levels of each protein, the OR of RA ranged from 0.09 (95% CI, 0.07 - 0.11; *P* = 2.10E-12) for AGER to 3.72 (95% CI, 1.74 - 7.96; *P* = 0.049) for EHBP1.

When the RA data is sourced from UKB, a total of 1,365 proteins are included in the replication analysis. After FDR adjustment, three proteins are significantly associated with the risk of RA. Among these, negative causal associations with RA were observed for AGER (OR, 0.98; 95% CI, 0.98 - 0.99; *P* = 1.82E-22), while positive causal associations were observed for two proteins: USP8 (OR, 1.01; 95% CI, 1.01 - 1.02; *P* = 0.031) and IDUA (OR, 1.01; 95% CI, 1.01 - 1.02; *P* = 0.036).

When the RA data is sourced from FinnGen, a total of 1,366 proteins are included in the replication analysis. After FDR adjustment, five proteins are significantly associated with the risk of RA. Among these, negative causal associations with RA were observed for three proteins (PPA2, PMEL, and AGER), while positive causal associations were observed for two proteins (F2 and JUND). Per SD increase in genetically predicted levels of each protein, the OR of RA ranged from 0.52 (95% CI, 0.37 - 0.74; P = 0.048) for AGER to 2.22 (95% CI, 1.45 - 3.40; *P* = 0.048) for JUND. S3 Table displays the full MR results.

### SMR analysis

When the eQTLs data were sourced from the eQTLGen consortium, five proteins from FinnGen passed all tests: DXO (β = -0.95; OR: 0.38; 95% CI: 0.30 - 0.49; *P* = 3.36E-15, *P*__HEIDI_ = 0.48), FCRL3 (β = 0.09; OR: 1.10; 95% CI: 1.05 - 1.14; **P* *= 1.22E-05, *P*__HEIDI_ = 0.30), KLRD1 (β = 0.17; OR: 1.18; 95% CI: 1.03 - 1.36; *P* = 0.015, *P*__HEIDI_ = 0.07), SWAP70 (β = -0.24; OR: 0.78; 95% CI: 0.68 - 0.91; *P* = 0.001, *P*__HEIDI_ = 0.40) and TNXB (β = 0.44; OR: 1.55; 95% CI: 1.43 - 1.68; *P* = 1.31E-27, *P*__HEIDI_ = NA). Seven proteins from UKB passed all tests: APOM (β = 0.77; OR: 2.17; 95% CI: 1.51 - 3.12; *P* = 2.88E-05, *P*__HEIDI_ = 0.26), DXO (β = -0.95; OR: 0.38; 95% CI: 0.30 - 0.49; *P* = 3.36E-15, *P*__HEIDI_ = 0.48), ERBB2 (β = -1.37; OR: 0.25; 95% CI: 0.14 - 0.46; *P* = 5.27E-06, *P*__HEIDI_ = 0.21), FCGR2B (β = 0.10; OR: 1.10; 95% CI: 1.02 - 1.20; *P* = 0.024, *P*__HEIDI_ = 0.21), IL1RN (β = -0.30; OR: 0.74; 95% CI: 0.56 - 0.99; *P* = 0.046, *P*__HEIDI_ = 0.11), SH2B3 (β = 0.30; OR: 1.35; 95% CI: 1.13 - 1.61; *P* = 0.001, *P*__HEIDI _= 0.05) and SUGP1 (β = 0.90; OR: 2.46; 95% CI: 1.41 - 4.29; *P* = 0.002, *P*__HEIDI_ = 0.18). Four proteins from Iceland passed all tests: TNFAIP3 (β = 0.86; OR: 2.36; 95% CI: 1.23 - 4.51; *P* = 0.009, *P*__HEIDI_ = NA), EHBP1 (β = 0.86; OR: 2.36; 95% CI: 1.23 - 4.51; *P* = 0.009, *P*__HEIDI_ = NA), USP8(β = -0.003; OR: 0.997; 95% CI: 0.995 - 0.998; *P* = 1.67E-04, *P*__HEIDI_ = 0.06) and IDUA (β = 0.003; OR: 1.003; 95% CI: 1.001753 - 1.004; *P* = 1.96E-05, *P*__HEIDI_ = 0.29) passed the SMR test.

**Fig 3:**
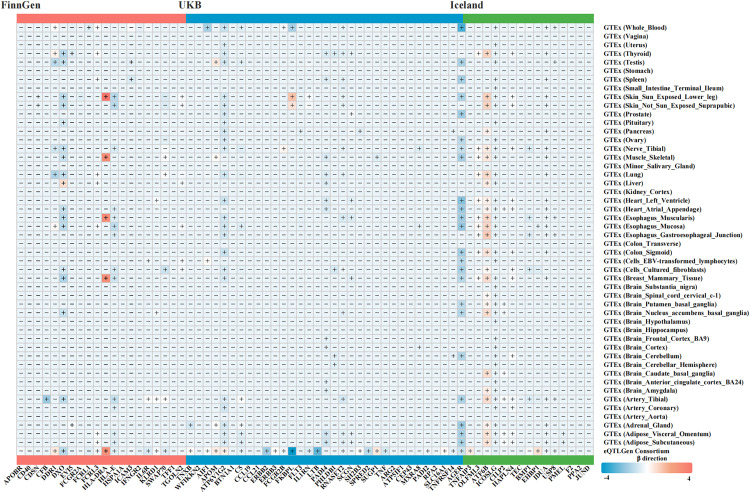
Heatmap of identified protein-coding genes associated with RA. Heatmap of the effect of plasma and tissue-specific protein-coding gene expression on RA risk for the identified proteins. The color represents the β estimators of SMR analysis, where blue indicates a decreased RA risk and orange indicates an increased RA risk per-SD increase in gene expression. Missing values marked with “-” indicate genes without effective eQTLs in the SMR analysis. RA, rheumatoid arthritis; SMR, summary-data-based mendelian randomization.

Due to the lack of relevant data for some proteins in the eQTLGen dataset, we supplemented the analysis with data from the GTEx database, which allowed us to obtain SMR results for an additional 13 proteins (WFIKKN2, TFF1, CDSN, CFB, MXRA8, BTN1A1, PHLDB1, SCGN, CDSN, HAPLN4, CILP2, OLFML3 and PMEL). Overall, using GTEx data, the analysis revealed that a total of 14 proteins from FinnGen (CDSN, DDR1, DXO, EVI5, FCRL3, HLA-DRA, HLA-E, ICAM3, IFNGR2, IL6R, KLRD1, SWAP70, TGOLN2 and WFIKKN2), 21 proteins from UKB (AIF1, APOM, ATP6V1G2, C5, CD40, DXO, EVI5, FCGR2B, FKBPL, FLT3M, HLA-DRA, ICAM3, IL1RN, PADI4, PHLDB1, RNASET2, SCGN, CDSN, MXRA8, RPA2 and TNFRSF14), and 12 proteins from Iceland (AGER, ATF6B, BMP1, CILP2, EHBP1, HAPLN4, ICOSLG, OLFML3, TEC, IDUA, USP8 and PMEL) showed SMR results consistent with the direction of the β value in at least one tissue. All results are detailed in Fig 3 and S4 Table.

### Colocalization analysis

For European populations, when colocalization analysis was conducted using plasma proteins from FinnGen sources and the RA data from Okada *et al.*, four proteins (FCGR2A, FCRL3, IL6R and TGOLN2) exhibited high colocalization support (P_H4_ ≥ 0.8) and four proteins (EVI5, FCRL1, HLA-DRA and SWAP70) exhibited medium colocalization support (P_H4_ = 0.5 - 0.8) for RA. When using plasma proteins from UKBPPP, six proteins (CD40, PADI4, ATP5IF1, CEP43, RPA2 and SUGP1) exhibited high colocalization support (P_H4_ ≥ 0.8) and four proteins (C5, EVI5, FLT3, PADI2 and IL1RN) exhibited medium colocalization support (PH4 = 0.5 - 0.8) for RA. When using plasma proteins from Iceland, eight proteins (AGER, TNFAIP3, ICOSLG, CILP2, HAPLN4, BMP1, EHBP1, and USP8) exhibited high colocalization support (P_H4_ ≥ 0.8) and six proteins (F2, TEC, FCRL1, PPA2, PMEL and JUND) exhibited medium colocalization support (P_H4_ = 0.5 - 0.8) for RA. For East Asian populations, two proteins (PILRA and PILRB) exhibited high colocalization support (P_H4_ ≥ 0.8), and two proteins (LRP11 and FCRL3) exhibited medium colocalization support (P_H4_ = 0.5 - 0.8) for RA.

**Fig 4:**
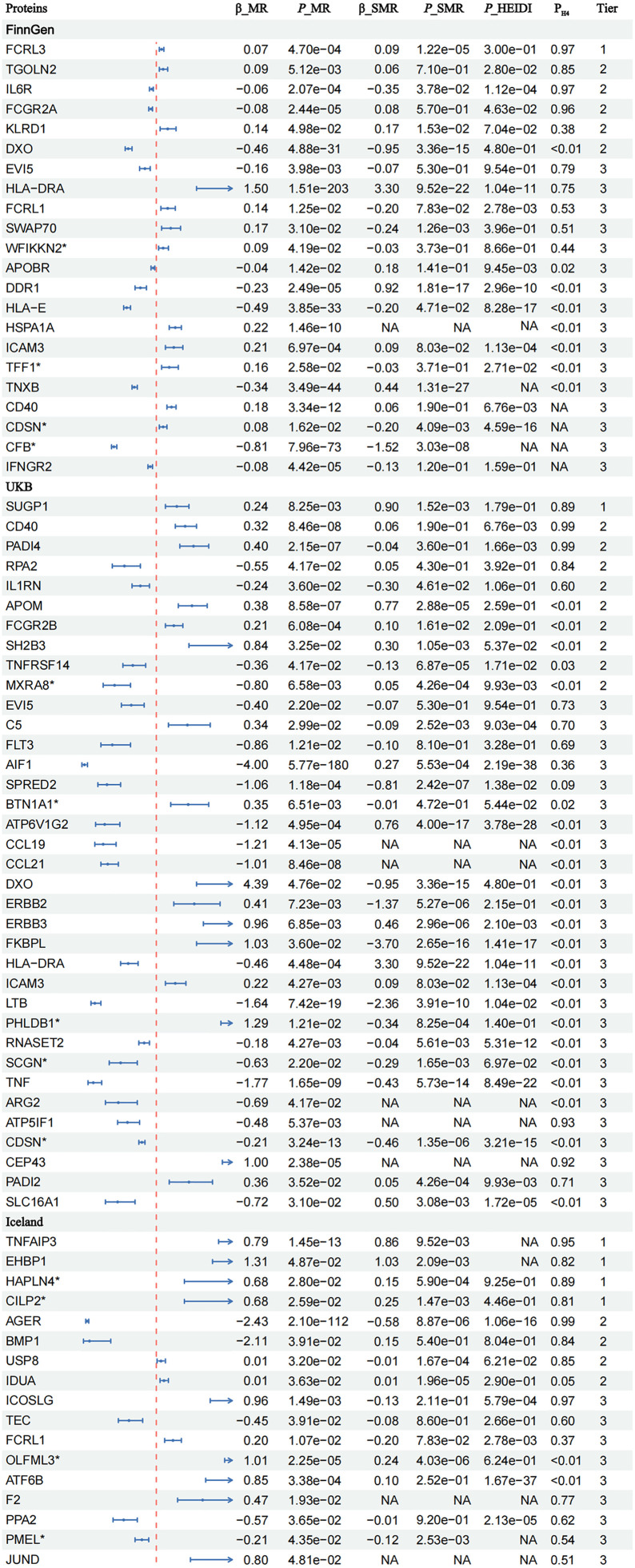
The forest plot presents the MR results, SMR results, and colocalization analysis for all proteins significantly associated with RA. Tier 1 indicates proteins that passed all tests (MR, SMR, HEIDI test, and colocalization analysis). Tier 2 represents proteins that passed the MR test and either the SMR or colocalization test. Tier 3 indicates proteins that only passed the MR test but did not pass the SMR or colocalization tests. Proteins on the left side of the dashed line are protective, while those on the right are risk proteins. NA indicates data was insufficient for analysis. MR, Mendelian randomization; SMR, summary-data-based mendelian randomization; RA, rheumatoid arthritis.

Fig 4 and S5 Table display the full results.

Indeed, among these proteins, FCRL3, SUGP1, TNFAIP3, EHBP1, HAPLN4 and CILP2 passed MR, colocalization, and SMR analyses simultaneously. These proteins were identified as core proteins and included in subsequent analyses.

### Enrichment analysis

**Fig 5:**
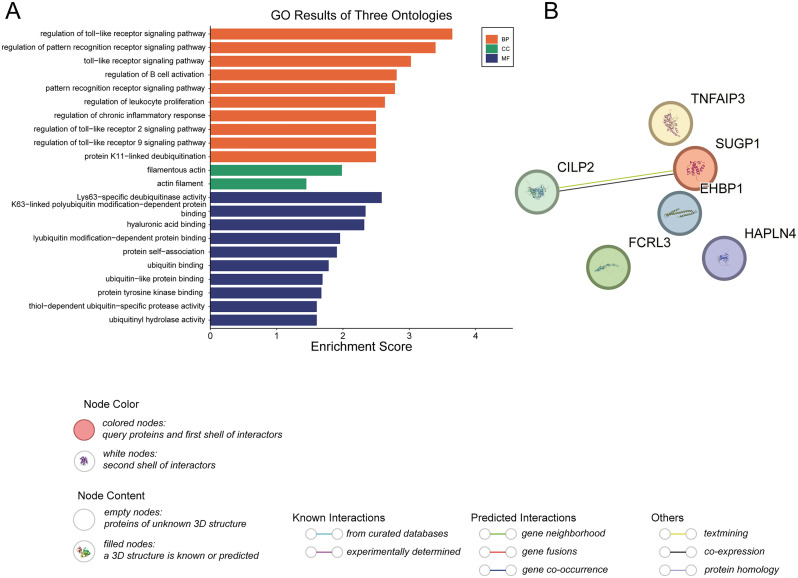
Enrichment and PPI analyses of the six key proteins (TNFAIP3, SUGP1, CILP2, EHBP1, FCRL3, HAPLN4). (A) GO function analysis histogram. BP is marked by dark cyan, CC by sienna, and MF by steel blue. The x-axis represents the enrichment score, and the y-axis denotes the GO terms. The top terms for BP, CC, and MF are displayed in ascending order of P-value. (B) Protein-protein interaction network analysis. Each node corresponds to a different protein, and the lines connecting the nodes represent potential interactions between these proteins. The different colors of the lines indicate varying levels of evidence for these interactions. GO, gene ontology; BP, biological process; CC, cell component; MF, molecular function; PPI, protein-protein interaction.

We performed GO analysis on the aforementioned six protein targets, including Biological Process (BP), Molecular Function (MF), and Cellular Component (CC), as shown in Fig 5. Notable BP terms include regulation of toll-like receptor signaling pathway, regulation of pattern recognition receptor signaling pathway, regulation of B cell activation, and regulation of chronic inflammatory response. CC enrichment analysis indicated that these proteins are primarily distributed in actin filaments. Significant MF terms include ubiquitinyl hydrolase activity, protein tyrosine kinase binding, and protein self-association. In addition, we performed KEGG pathway enrichment analysis. The results showed that these proteins were significantly enriched in several inflammation- and immune-related pathways, including the IL-17 signaling pathway, NF-κB signaling pathway, and TNF signaling pathway (S1 Fig). These pathways are well-established components of the pathogenesis of RA, suggesting that the identified proteins may be involved in key inflammatory cascades. Other significantly enriched pathways included the NOD-like receptor signaling pathway, necroptosis, and Epstein–Barr virus infection, further indicating that immune dysregulation and viral triggers may contribute to the disease mechanism.The biological processes, molecular functions, and signaling pathways involving these proteins are closely associated with the pathogenesis of RA [52–56].

### PPI analyses

Based on the current evidence, there is little interaction among the six proteins mentioned above (Fig 5B). CILP2 interacts with SUGP1, with supporting evidence from text mining and/or co-expression data. The remaining proteins are independent nodes with no apparent interactions with the other proteins. This suggests that, in the context of RA, these proteins may influence cellular behavior either independently or through interactions with other proteins outside of these six, thereby impacting the pathophysiology of RA.

To further explore potential indirect associations, we performed an expanded PPI analysis using the GeneMANIA platform. This analysis incorporated 26 proteins, including the six identified hub proteins. The resulting network revealed extensive functional associations among these proteins, including physical interactions, co-expression, genetic interactions, and pathway co-occurrence (S2A Fig). Notably, nodes such as HAPLN4, CILP2, and TNFAIP3 exhibited high degrees of connectivity, suggesting their potential roles as key regulatory hubs within the network.

Subsequent GO enrichment analysis demonstrated that these core genes are primarily involved in immune-related BP, such as the toll-like receptor signaling pathway, NF-κB signaling, and B cell activation (S2B Fig). In terms of CC, the genes were enriched in structures such as the spliceosomal complex, membrane rafts, and nucleosomes. For MF, enrichment was observed in protein modification-related activities including ubiquitin ligase binding and deubiquitinase activity. Together, these findings suggest that the candidate genes may contribute to RA pathogenesis by regulating immune-inflammatory signaling and maintaining protein homeostasis.

### Integration and differential analysis of bulk transcriptomic datasets

The differential analysis results of the bulk transcriptomics data are shown in S6 and S7 Tables. The integrated expression matrix contains transcriptomic expression data from a total of 843 blood/cell samples (666 RA patients and 177 healthy controls) or 40 synovial samples (33 RA synovial samples and 7 healthy controls). The differential analysis results indicate that compared to healthy controls, CILP2 is upregulated in the peripheral blood of RA patients, while HAPLN4, FCRL3, EHBP1, and TNFAIP3 are downregulated (S6 Table). Except for CILP2, the expression trends of the other genes are inconsistent with the colocalization analysis results. Interestingly, compared to healthy controls, TNFAIP3 and FCRL3 are upregulated in the synovial tissue of RA patients, while EHBP1, CILP2, and HAPLN4 show no significant difference in expression (S7 Table). The tissue-specific expression of TNFAIP3 and FCRL3 suggests that they may have different functions and regulatory mechanisms in different tissues.

### ROC analysis

**Fig 6:**
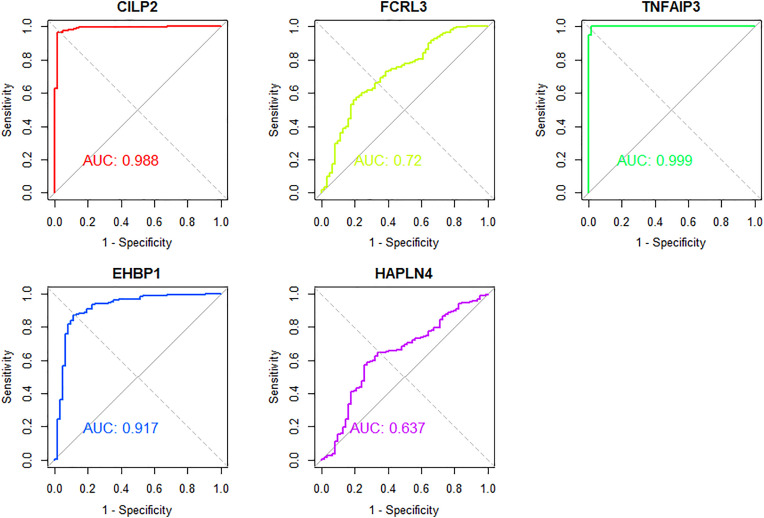
ROC curves for TNFAIP3, CILP2, EHBP1, FCRL3 and HAPLN4. The x-axis represents the false positive rate, also known as 1-specificity, while the y-axis represents the true positive rate or sensitivity. The area under the curve is used to quantify the overall ability of the model to discriminate between categories. The diagonal line from (0,0) to (1,1) serves as a reference, indicating a model with no discriminatory power (AUC = 0.5). Curves closer to the upper left corner indicate higher overall accuracy and effectiveness of the model in correctly classifying results. AUC, area under the curve.ROC, Receiver operating characteristic curve.

Based on the integrated transcriptomic expression matrix (blood), ROC analysis was performed for the six proteins supported by colocalization. SUGP1 was not present in the transcriptomic expression matrix and was therefore excluded. As shown in Fig 6, the AUC value for FCRL3 ranges between 0.7 and 0.8, while the AUC values for CILP2, TNFAIP3, and EHBP1 range between 0.9 and 1. The AUC value for HAPLN4 is below 0.7 and was therefore not considered. These findings suggest that CILP2, TNFAIP3, and EHBP1 exhibit strong discriminatory performance in distinguishing between RA and non-RA states, highlighting their potential as RA biomarkers.

### Random forest modeling

To further evaluate the predictive performance of the selected protein panel (CILP2, FCRL3, TNFAIP3, EHBP1, and HAPLN4), we applied a random forest classification model. The model demonstrated excellent classification accuracy in distinguishing RA patients from healthy controls, with the ROC curve approaching the top-left corner and an AUC of approximately 1.00, indicating near-perfect discriminative ability. SUGP1 was excluded from the analysis due to the absence of expression data in the transcriptomic dataset.

Feature importance analysis based on the mean decrease in Gini index revealed that TNFAIP3 contributed the most information to the model, followed by CILP2 and EHBP1, while FCRL3 and HAPLN4 had relatively smaller contributions. These results highlight the dominant role of TNFAIP3 in the predictive model and suggest its potential utility as a key biomarker for RA diagnosis or risk stratification. (S3 Fig).

### Drug prediction, molecular docking, and molecular dynamics simulations

**Fig 7:**
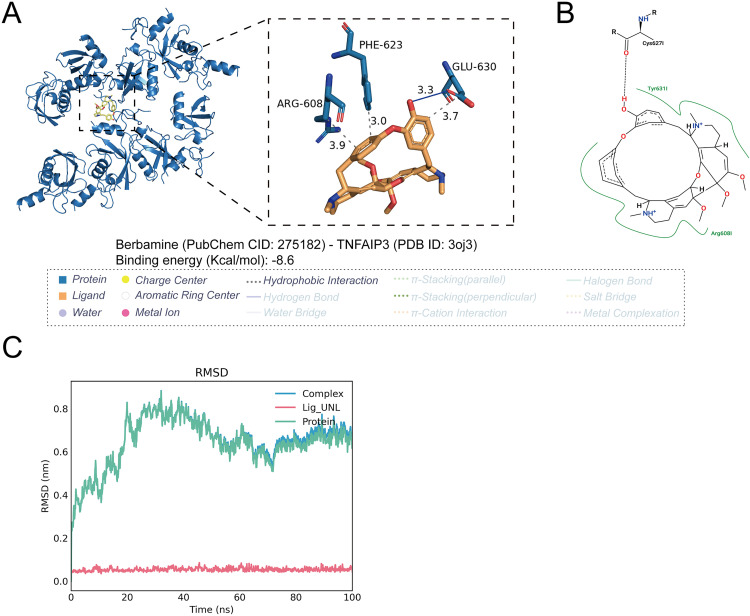
Molecular docking models and molecular dynamics simulations of proteins, ligands, and protein-ligand complexes. (A left) Macroscopic 3D molecular docking model of TNFAIP3-Berbamine. (A right) Microscopic 3D molecular docking model of TNFAIP3-Berbamine. (B) 2D molecular docking model of TNFAIP3-Berbamine. (C) Molecular Dynamics Simulations of TNFAIP3 Proteins, Berbamine Ligands, and TNFAIP3-Berbamine Complexes. The X-axis represents simulation time in nanoseconds (ns). The Y-axis represents the RMSD values in nanometers (nm). The blue curve (Complex) denotes the RMSD changes of the entire complex. The red curve (Lig_UNL) represents the RMSD changes of the ligand. The green curve (Protein) indicates the RMSD changes of the protein.

Based on predictions from Enrichr-DsigDB, potential upstream targeting drugs for TNFAIP3, EHBP1, FCRL3, SUGP1, HAPLN4, and CILP2 were identified (S8 Table). Molecular docking results reveal that Berbamine (PubChem CID: 275182) binds to TNFAIP3 (PDB ID: 3oj3) with a binding energy of -8.6 kcal/mol, exceeding the threshold of -7.2 kcal/mol, indicating strong binding affinity (Fig 7A, B). In addition, molecular docking analyses were performed for EHBP1, FCRL3, SUGP1, HAPLN4, and CILP2 with their corresponding compounds (S4A–E Fig). The results indicated that these proteins also exhibit strong binding affinities with their respective small-molecule ligands.

Additionally, molecular dynamics simulations show that the RMSD trends for the Berbamine-TNFAIP3 protein-ligand complex and the protein monomer are very similar, suggesting that conformational changes in the protein significantly impact the stability of the complex throughout the simulation (Fig 7C). For the Berbamine-TNFAIP3 complex, the RMSD stabilizes after approximately 55 nanoseconds, indicating that the structure of both the protein and the complex reaches a stable conformation. Throughout the simulation, the ligand’s RMSD remains within a very low range (approximately 0.1 to 0.1 nm) with minimal fluctuations, suggesting that the ligand maintains a relatively stable position and conformation without significant drift or conformational changes. These results indicate that the binding of TNFAIP3 to Berbamine is relatively stable throughout the molecular dynamics simulations.

### Phe-WAS

In the analyses above, CILP2 passed all tests. Therefore, we conducted a Phe-WAS on it. S9 Table displays the result summary of the analyses of CILP2 in relation to other disease outcomes. We observed potential causal associations between CILP2 and 170 phenotypes. The most significant negative causal effect was observed in association with intervertebral disc disorders (β = -0.38; OR: 0.68; 95% CI: 0.55 - 0.83; *P* = 1.39E-04), and the most significantly positive causal effect was observed in association with ulcerative rectosigmoiditis (β = 1.38; OR: 3.99; 95% CI:2.03 - 8.83; *P* = 6.04E-05).

## Discussion

We identified 68 proteins with significant causal relationships with RA. Since the destructive process in joints is irreversible, early diagnosis and treatment are crucial for improving the prognosis of patients with RA. In this study, we identified large number of plasma proteins causally associated with RA. These proteins not only provide new biological insights for the early diagnosis of RA but also broaden potential directions for future therapeutic strategies.

Previous studies have explored the association between FCRL3 and RA, revealing significant differences in this relationship across different ethnic groups. Specifically, studies have found that the expression of the *FCRL3* gene is significantly associated with the risk of RA in Chinese and Japanese populations, while the results are inconsistent in European populations [57–62]. The reasons behind this ethnic variability may be complex and diverse, including gene-gene interactions, genetic background diversity, the influence of comorbid diseases (particularly autoimmune diseases), and the combined effects of lifestyle and environmental factors [63,64]. The GWAS data used in our study are derived from European populations, and the results show that variations in the *FCRL3* gene significantly increase the risk of RA in European populations. This finding provides new evidence for the relationship between FCRL3 and the risk of RA. Furthermore, previous studies suggested that FCRL3 can not only independently increase the risk of RA, but also further elevate the risk in the presence of a higher frequency of the *HLA-DRB1* gene [65,66]. A recent study further revealed the connection between FCRL3 and RA through expression quantitative trait locus (eQTL) analysis. This study found that the *FCRL3* gene is regulated by the same cis-eQTL effect in CD4 + T cells and B cells, leading to an increased risk of RA [67]. This finding is consistent with our SMR analysis results. Whether based on data from the eQTLgen consortium or the GTEx project, we observed that *FCRL3* gene expression can significantly increase the risk of RA (S3 Table). In addition, we performed MR and colocalization analysis using GWAS data on plasma proteins. This analysis effectively reduced the impact of potential confounding factors, providing protein-level evidence that further supports the causal relationship between FCRL3 and RA, suggesting that targeting FCRL3 may help improve the prognosis of RA patients.

TNFAIP3 (also known as A20) is a deubiquitinating enzyme that primarily regulates key molecules in the nuclear factor-kappa B (NF-κB) signaling pathway (such as RIP1, RIP2, TRAF6, and MALT1), thereby inhibiting NF-κB-dependent gene expression and exerting its anti-inflammatory effects [68]. Although extensive research has explored the function of TNFAIP3, its mechanism of action remains incomplete and findings are inconsistent. For instance, one study found that TNFAIP3-deficient mice exhibited significantly elevated serum levels of inflammatory cytokines, accompanied by ankle synovitis, monocyte infiltration, and damage to cartilage and bone tissue [69]. Interestingly, these pathological features were ameliorated following injection of an rAAV6 virus containing the TNFAIP3 gene, suggesting that TNFAIP3 plays a crucial role in controlling inflammation and tissue damage [70]. However, recent research indicates that A20 knockout mice displayed only osteoporosis, without elevated levels of inflammatory cytokines or related features of inflammatory arthritis [71]. These contradictory results suggest that the function of TNFAIP3 may be influenced by specific experimental conditions or models. While animal model studies suggest a potential protective role for TNFAIP3, this effect has not been fully corroborated in human clinical studies. An early GWAS meta-analysis involving 15,855 subjects demonstrated that TNFAIP3, CD40, and PADI4 were significantly associated with susceptibility to RA [72]. This finding has been supported by subsequent studies, reinforcing the potential role of TNFAIP3 as a risk factor for RA [73,74]. Furthermore, recent studies have revealed the functional scope of TNFAIP3, showing that it is not only expressed in immune cells but also directly involved in the pathological process of RA by regulating cell proliferation and activation in non-immune cells, such as synovial fibroblasts in RA patients [75]. These results suggest that TNFAIP3 may play multiple roles in the onset and progression of RA. This study employed MR analysis of GWAS data, providing additional evidence of the harmful effects of TNFAIP3 in RA patients. Nonetheless, the specific mechanisms through which TNFAIP3 operates in RA require further investigation to offer clearer insights for future therapeutic strategies.

Our study demonstrates a significant association between CILP2 and the risk of RA. CILP2, a cartilage intermediate layer protein, expressed most abundantly in cartilaginous tissues, and plays a role in the structure and function of non-chondral tissue extracellular matrix. Previous studies have focused on the potential role of CILP2 in osteoarthritis. An early basic study utilizing an osteoarthritis mouse model found that as cartilage degraded, the expression and protein levels of the CILP2 gene were markedly downregulated. This suggested that CILP2 might be a key factor contributing to cartilage erosion and degeneration [76]. However, this results are not entirely consistent with clinical observations. An observational study comparing volleyball players across different age groups showed that CILP2 levels were significantly elevated in adult athletes compared to younger ones. This suggested that increased CILP2 levels may be linked to a higher risk of osteoarthritis, particularly in the degenerative changes observed in the knee joint [77,78]. Moreover, a proteomics study confirmed that *CILP2* gene expression was significantly elevated in the cartilage of osteoarthritis patients compared to non-osteoarthritis individuals, indicating that CILP2 could potentially serve as a predictive marker for the onset of osteoarthritis [78]. Another study, which analyzed osteoarthritis patients who underwent total knee replacement surgery, found significantly elevated CILP2 levels in the joint cartilage post-surgery. Transmission electron microscopy further revealed severe cartilage cell damage in these patients, reinforcing the potential role of CILP2 in cartilage injury [79]. RA is a systemic autoimmune disease characterized by synovitis and the destruction of joint cartilage, with some overlap in pathological mechanisms between RA and OA, particularly in the processes that lead to cartilage damage [80]. Despite these similarities, systematic research on the specific expression and functional mechanisms of CILP2 in RA is currently lacking. In this study, our MR analysis revealed a significant correlation between CILP2 and the risk of RA, suggesting that CILP2 may play a role in the onset and progression of RA. However, this association requires further validation through both basic and clinical research.

In our study, we identified several proteins, including HAPLN4, SUGP1, and EHBP1, that had not been previously reported in the context of RA. While previous research has largely focused on the association between HAPLN4 and psychiatric disorders [81–84], its involvement in joint diseases remains understudied. An early genome-wide expression study on limb cartilage from 13.5-day embryonic mice and growth plate cartilage from 5-week-old mice demonstrated significant abnormalities in HAPLN4 gene expression within the growth plate tissues of mucopolysaccharidosis mouse models [85]. These findings suggest that HAPLN4 may disrupt cartilage development and contribute to the early pathological processes of joint diseases. However, the study is limited to animal models and does not report the collagen to keratin ratio differences within this tissue. Future studies are needed to further clarify the specific role of HAPLN4 in human subjects. Furthermore, although our study utilized proteomic data from various populations, HAPLN4 has not been validated in Asian populations. Therefore, subsequent research should explore the impacts of geographic location, linguistic groups, and cultural factors on its function. In addition, previous research has only identified potential associations between SUGP1 and EHBP1 with lipid levels [86,87], and no detailed studies have explored their relationship with RA risk. Our research showed that both SUGP1 and EHBP1 are significantly associated with an increased risk of RA. Notably, further colocalization analysis and SMR analysis reinforced this association, indicating that these two proteins may play pivotal roles in the pathogenesis of RA and represent potential therapeutic targets for drug development. These new findings further expand the molecular network of RA and provide valuable insights for the treatment and intervention strategies of this disease.

Previously, two studies investigated potential drug targets for RA using MR analysis [88,89]. Our study, however, offers several notable advantages: 1) We utilized the largest GWAS data for plasma proteomics to date as exposure variables, combined with multiple RA cohorts as outcome variables for MR analysis. This approach significantly enhanced statistical power, allowing for more precise and reliable estimates of potential protein targets for RA; 2) Through systematic analysis, we preliminarily identified 68 plasma proteins with significant causal relationships with RA, greatly expanding the known spectrum of RA-related proteins. The identification of these proteins not only provides new insights into the molecular mechanisms of RA but also points to potential intervention directions for future drug development; 3) Compared to previous studies, we adopted more comprehensive statistical analysis methods, including MR, SMR, colocalization analysis, and single-cell analysis to ensure the robustness of our results. Through these multidimensional analyses, we ultimately identified six plasma proteins with the highest potential as drug targets, providing crucial evidence for precision therapy in RA; 4) In our study, not only did we utilize traditional MR analysis, but we also introduced more detailed single-cell analysis and molecular docking techniques for the first time.This innovative approach allowed us to further validate the functions of the identified proteins and their potential relationships with RA at the cellular and structural levels, enhancing the biological interpretability of our results. Moreover, the molecular docking analysis further explored the interactions between these target proteins and small-molecule drugs, providing practical preliminary data support for future drug development.

However, our study has some limitations. 1) The GWAS data used in the study were all from European and East Asian populations, indicating that the results of this study may not be applicable to individuals of other ancestries; 2) Due to insufficient available data and the heavy multiple testing burden, some proteins could not be completely analyzed, potentially resulting in the loss of opportunities for further evaluation of these proteins. However, this strategy suits one of this study’s aims, which is to discover proteins strongly associated with RA; 3) In SMR analysis, the majority of the data is derived from blood samples, with only a few from the facet joint. This may result in slight biases due to the specific expression of genes; 4) In our study, some proteins associated with RA exhibited inconsistent MR results across different datasets, and this inconsistency could be a limitation of sampling. To avoid false positive results, only proteins that passed MR, colocalization, and SMR analyses simultaneously were included in subsequent analyses, but the limitation of sampling could remain. We look forward to future data that can elucidate the causal relationship between these proteins and RA; 5) Due to the admixture of populations with different genetic backgrounds in the GWAS data sources, the admixture effect and threshold influence on the study samples may be difficult to avoid. To address this limitation, we employed the most stringent criteria when selecting instrumental variables (*P*_1_ = 5E-08, *r*^2^ = 0.001, kb = 10000, cis_win = 1000) to avoid biases introduced by weak instruments. For the MR analysis results, we applied false discovery rate (FDR) correction to mitigate the risk of false positives. Additionally, we utilized different protein and RA data sources and conducted intersection analyses based on the results to avoid the confounding effects of heterogeneous samples; 6) as these associations were established through in silico analyses, the underlying mechanisms are not clear and require further validation through animal studies and population-based research.

In conclusion, we identified a total of 68 plasma proteins with causal associations to RA, and after multiple testing and analyses, six proteins (FCRL3, SUGP1, TNFAIP3, EHBP1, HAPLN4, and CILP2) were recognized as having the highest potential as drug targets. Subsequent analyses further validated that targeting these proteins could help improve patient prognosis. We look forward to more research in the future to reveal the specific roles of these proteins in RA patients.

## Supporting information

S1 Table Details of the data sources used in this study. (S1_Table.XLSX)

S2 Table Detailed information on the RA-related bulk transcriptomic datasets selected from the GEO database. (S2_Table.XLSX)

S3 Table Full MR result of plasma proteins with significant causal relationships to rheumatoid arthritis. (S3_Table.XLSX)

S4 Table Full SMR result of plasma proteins with significant causal relationships to rheumatoid arthritis. (S4_Table.XLSX)

S5 Table Full Colocalization result of plasma proteins with significant causal relationships to rheumatoid arthritis. (S5_Table.XLSX)

S6 Table Differential expression results returned by the Rank_in algorithm (blood/ blood cells). (S6_Table.XLSX)

S7 Table Differential expression results returned by the Rank_in algorithm (synovial membrane). (S7_Table.XLSX)

S8 Table The potential upstream targeted drugs predicted by Enrichr-DsigDB for TNFAIP3, SUGP1, CILP2, EHBP1, FCRL3 and HAPLN4. (S8_Table.XLSX)

S9 Table Full result of Phe-WAS analysis of associations between CILP2 and other disease outcomes. (S9_Table.XLSX)

S1 Fig KEGG pathway enrichment analysis of the six hub proteins. Dot plot visualization of the top enriched KEGG pathways. The x-axis represents the enrichment score (−log₁₀ p-value), and the y-axis lists the names of enriched pathways. Dot size indicates the number of genes (Count) involved in each pathway, and dot color represents the corresponding p-value.
(S1_Fig.TIF)

S2 Fig Construction and enrichment analysis of the extended protein–protein interaction (PPI) network. (A) PPI network constructed based on the candidate core genes. Node size indicates degree of connectivity. Edges represent different types of interactions, including physical interactions (red), predicted interactions (orange), genetic interactions (green), co-expression (pink), pathway (blue), co-localization (cyan), and shared protein domains (yellow). (B) Gene Ontology (GO) enrichment analysis results for the candidate genes, shown across the three main ontologies: biological process (BP, orange), cellular component (CC, green), and molecular function (MF, blue). The x-axis represents the enrichment score.
(S2_Fig.TIF)

S3 Fig Random forest analysis based on the selected protein panel. (A) ROC curve showing the classification performance of the random forest model. (B) Feature importance ranked by mean decrease in Gini index.
(S3_Fig.TIF)

S4 Fig Molecular docking models of candidate small-molecule compounds with predicted target proteins. (A) Docking model of Diethylnitrosamine with HAPLN4. (B) Docking model of Torcetrapib with EHBP1. (C) Docking model of Amiodarone with FCRL3. (D) Docking model of Cephaeline with SUGP1. (E) Docking model of Valproic acid with CILP2.The left panels show the overall structure of each protein-ligand complex, while the right panels present close-up views of the binding pockets, highlighting key interacting residues and hydrogen bond distances (in Å). Binding energies (kcal/mol) are indicated for each complex.
(S4_Fig.TIF)

S1 File Parameter file used for the energy minimization step in GROMACS molecular dynamics simulations. This file applies the steepest descent algorithm with a maximum of 50,000 steps and a convergence criterion of 1000 kJ/mol/nm, ensuring removal of steric clashes and relaxation of initial structures before equilibration. This file is in plain text format but is intended for use with GROMACS; users should open it with a text editor or within the GROMACS environment.
(MDP)

S2 File Parameter file used for the NPT equilibration phase under constant pressure and temperature. It uses the V-rescale thermostat (300 K) and Parrinello-Rahman barostat (1.0 bar), with a time step of 2 fs and constraints applied via the LINCS algorithm. This setup stabilizes pressure and density before the production MD run. This file is in plain text format but is intended for use with GROMACS; users should open it with a text editor or within the GROMACS environment.
(MDP)

S3 File Parameter file used for the production molecular dynamics simulation. It defines a 50 ns simulation using a 2 fs time step, with temperature and pressure maintained using V-rescale thermostat and Parrinello-Rahman barostat. The file includes constraints, neighbor list updates, and energy output settings for trajectory collection and analysis. This file is in plain text format but is intended for use with GROMACS; users should open it with a text editor or within the GROMACS environment.
(MDP)

S4 File Raw gene expression data of samples used for ROC analysis. (S4_File.CSV)

S5 File Raw group classification data of samples used for ROC analysis. (S5_File.CSV)

S6 File Raw gene expression data of samples used for random forest analysis. (S6_File.CSV)

S7 File Raw group classification data of samples used for random forest analysis. (S7_File.CSV)

S1 Code Core code for MR analysis. (S1_Code.DOCX)

S2 Code Core code for colocalization analysis. (S2_Code.DOCX)

S3 Code Core code for SMR analysis. (S3_Code.DOCX)

S4 Code Core code for ROC analysis. (S4_Code.DOCX)

S5 Code Core code for random forest models. (S5_Code.DOCX)
