# Supplementary figures and images for: Exploration of potential novel drug targets for rheumatoid arthritis by plasma proteome screening
Source: PLoS Comput Biol. 2025 Sep 25;21(9):e1013333. doi: 10.1371/journal.pcbi.1013333 (PMC12463240; doi:10.1371/journal.pcbi.1013333)

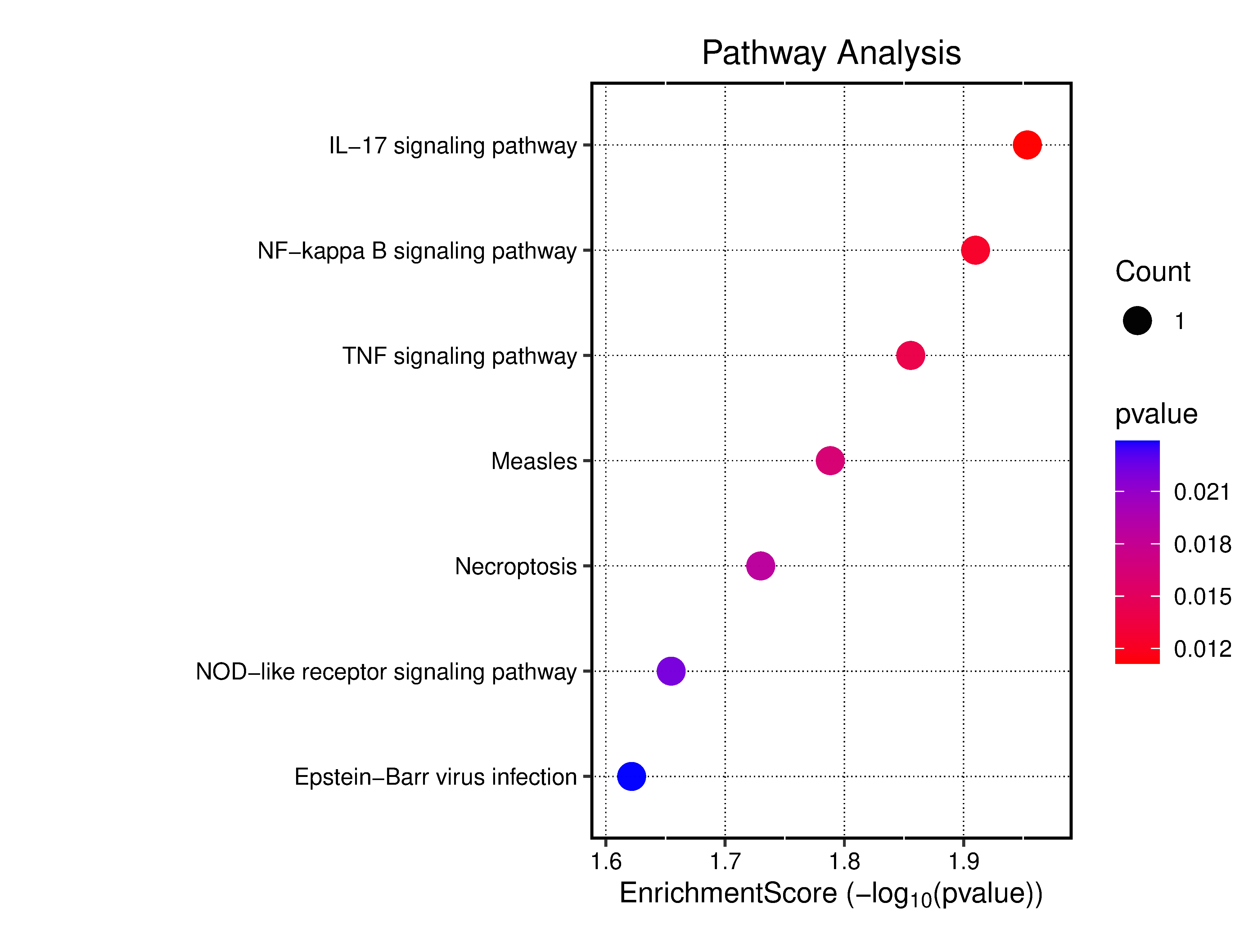

Supplement: S1 Fig — Dot plot visualization of the top enriched KEGG pathways. The x-axis represents the enrichment score (−log₁₀ p-value), and the y-axis lists the names of enriched pathways. Dot size indicates the number of genes (Count) involved in each pathway, and dot color represents the corresponding p-value. (S1_Fig.TIF) [file pcbi.1013333.s010.tif]

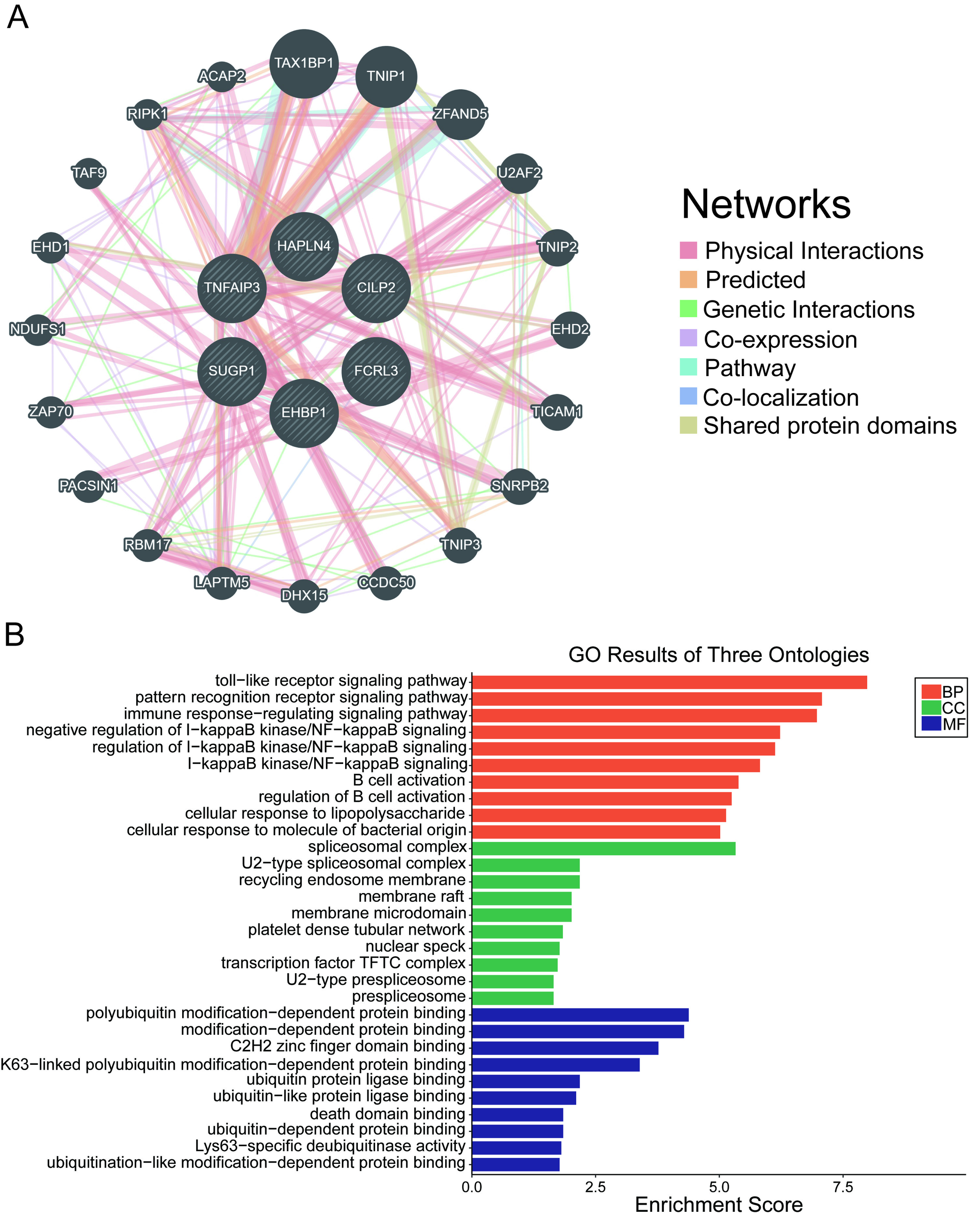

Supplement: S2 Fig — (A) PPI network constructed based on the candidate core genes. Node size indicates degree of connectivity. Edges represent different types of interactions, including physical interactions (red), predicted interactions (orange), genetic interactions (green), co-expression (pink), pathway (blue), co-localization (cyan), and shared protein domains (yellow). (B) Gene Ontology (GO) enrichment analysis results for the candidate genes, shown across the three main ontologies: biological process (BP, orange), cellular component (CC, green), and molecular function (MF, blue). The x-axis represents the enrichment score. (S2_Fig.TIF) [file pcbi.1013333.s011.tif]

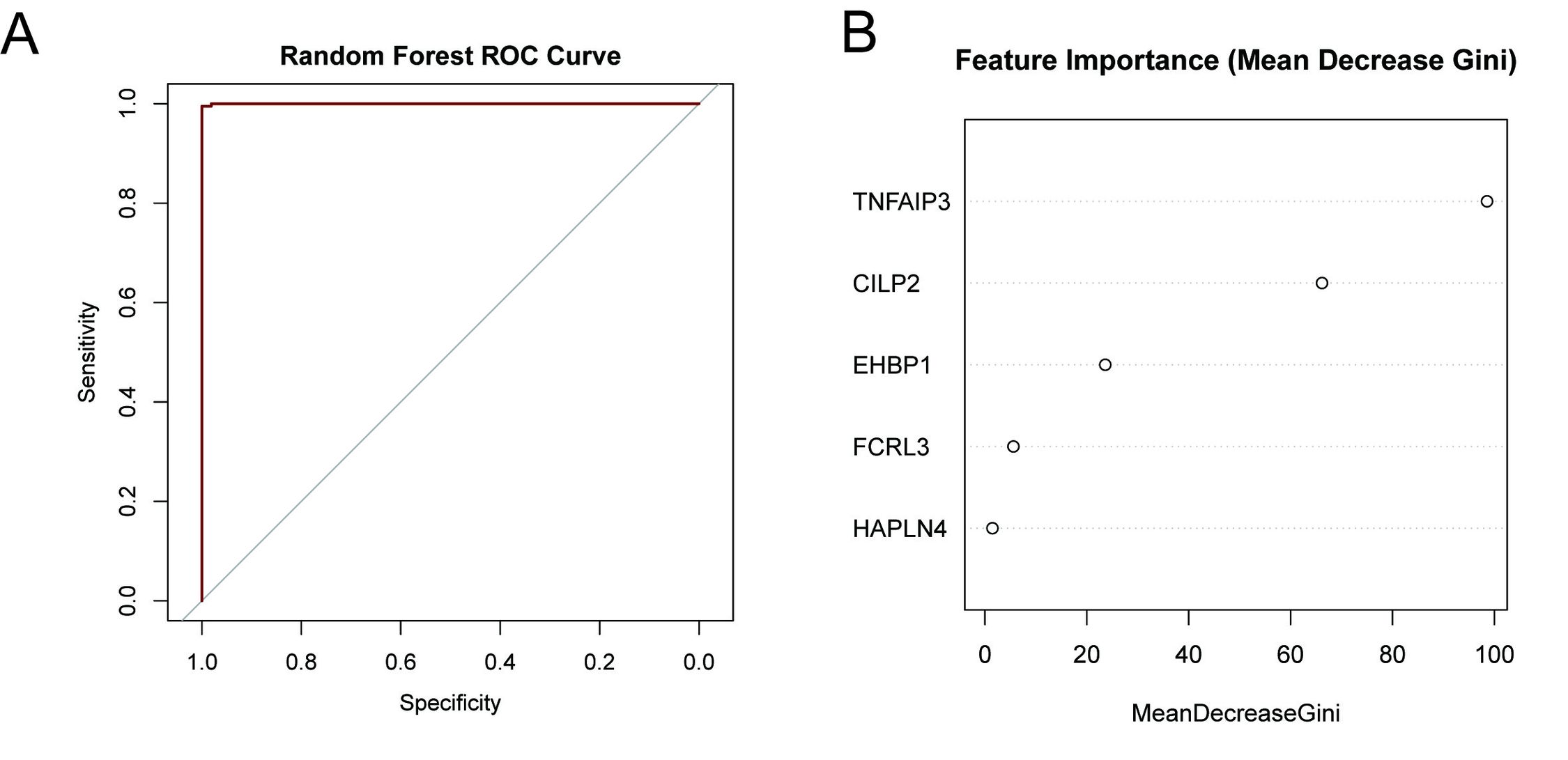

Supplement: S3 Fig — (A) ROC curve showing the classification performance of the random forest model. (B) Feature importance ranked by mean decrease in Gini index. (S3_Fig.TIF) [file pcbi.1013333.s012.tif]

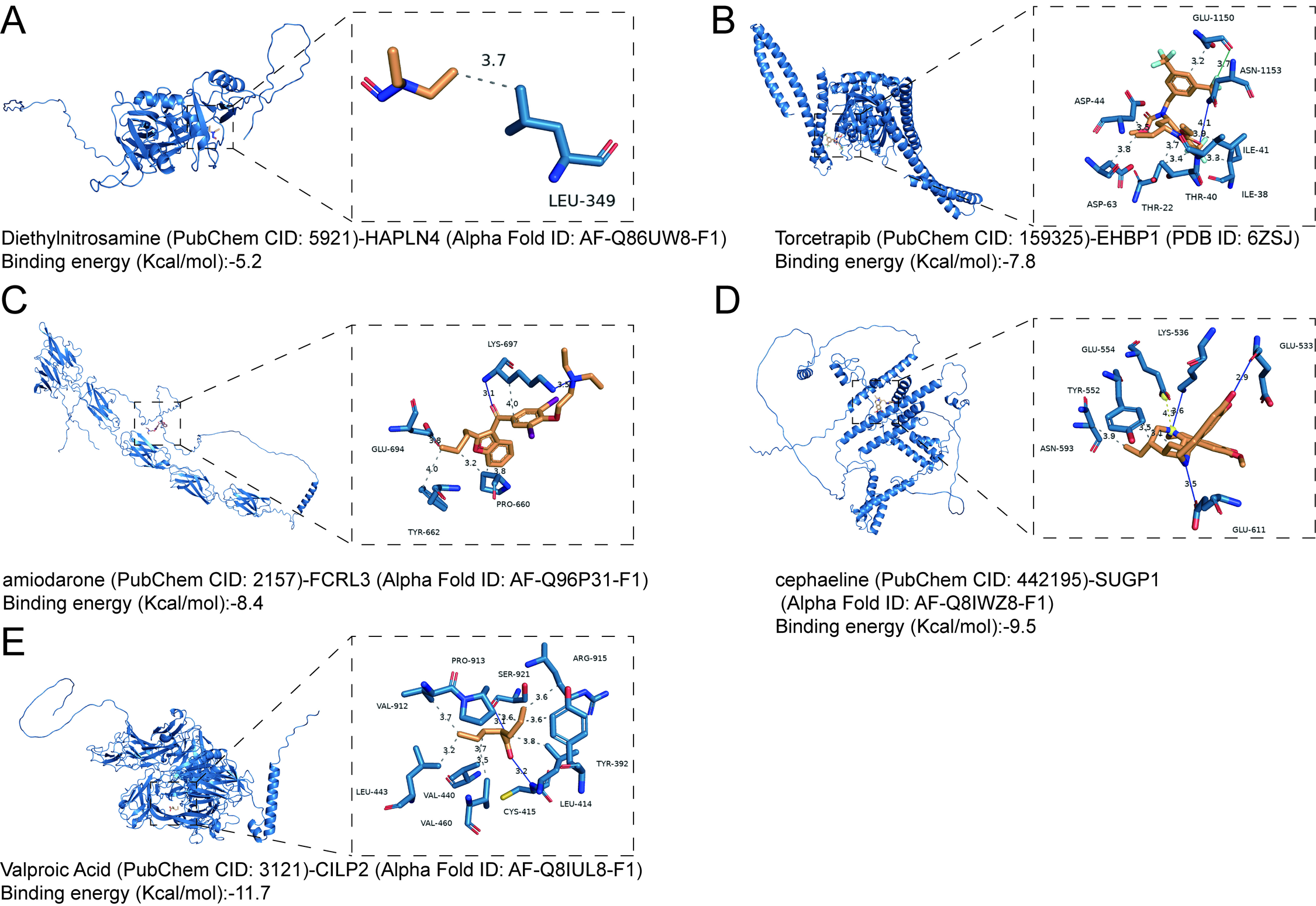

Supplement: S4 Fig — (A) Docking model of Diethylnitrosamine with HAPLN4. (B) Docking model of Torcetrapib with EHBP1. (C) Docking model of Amiodarone with FCRL3. (D) Docking model of Cephaeline with SUGP1. (E) Docking model of Valproic acid with CILP2.The left panels show the overall structure of each protein-ligand complex, while the right panels present close-up views of the binding pockets, highlighting key interacting residues and hydrogen bond distances (in Å). Binding energies (kcal/mol) are indicated for each complex. (S4_Fig.TIF) [file pcbi.1013333.s013.tif]
