# Supplementary material for: Exploration of potential novel drug targets for rheumatoid arthritis by plasma proteome screening
Source: PLoS Comput Biol. 2025 Sep 25;21(9):e1013333. doi: 10.1371/journal.pcbi.1013333 (PMC12463240; doi:10.1371/journal.pcbi.1013333)
Supplement: S1 Code — (S1_Code.DOCX) [file pcbi.1013333.s021.docx]

**S1 Code. Core code for MR analysis**

#run mr function,remove some snps

mr_fun <- function(id_exposures, id_outcomes, p1,

method_list = c("mr_ivw","mr_egger_regression","mr_weighted_median", "mr_weighted_mode","mr_wald_ratio"),

rm_snps=NULL){

#1.read exposure data

exposure_data <- TwoSampleMR::extract_instruments(id_exposures, p1=p1, r2=0.1, kb=10000)

#remove some snps

if(is.null(rm_snps)){

exposure_data <- subset(exposure_data, !SNP %in% rm_snps)

}

#2.read outcome data

#outcome_data <- TwoSampleMR::extract_outcome_data(exposure_data$SNP, outcomes = id_outcomes, proxies = F)

TwoSampleMR::read_outcome_data(id_outcomes, exposure_data$SNP)

#3.harmonise data

harmonise_data <- TwoSampleMR::harmonise_data(exposure_data, outcome_data)

#4.run mr analysis

mr_res <- TwoSampleMR::mr(harmonise_data, method_list = method_list)

#5. run presso

#presso_res <- MRPRESSO::mr_presso(harmonise_data$beta.outcome, harmonise_data$beta.exposure, harmonise_data$se.outcome, harmonise_data$se.exposure)

return(mr_res)

}

#run batch mr

mr_res <- mr_fun("exposure_data.csv", " outcome_data.csv", p1 = 5e-08)
