# Supplementary material for: Exploration of potential novel drug targets for rheumatoid arthritis by plasma proteome screening
Source: PLoS Comput Biol. 2025 Sep 25;21(9):e1013333. doi: 10.1371/journal.pcbi.1013333 (PMC12463240; doi:10.1371/journal.pcbi.1013333)
Supplement: S2 Code — (S2_Code.DOCX) [file pcbi.1013333.s022.docx]

**S2 Code. Core code for colocalization analysis**

#Use coloc::coloc.abf for colocalization

qtl_file = ""

gwas_file = ""

qtl <- data.table::fread(qtl_file, sep="auto", header = T)

gwas <- data.table::fread(gwas_file, sep="auto", header = T)

dup <- duplicated(qtl$SNP)

if(any(dup)){

qtl <- qtl[!dup,]

}

dup <- duplicated(gwas$SNP)

if(any(dup)){

gwas <- gwas[!dup,]

}

if(is.character(qtl$pval)){

qtl$pval <- gsub("e", "E", qtl$pval)

qtl$pval <- as.numeric(qtl$pval)

}

if(is.character(gwas$pval)){

gwas$pval <- gsub("e", "E", gwas$pval)

gwas$pval <- as.numeric(gwas$pval)

}

qtl <- subset(qtl, pval > 0)

gwas <- subset(gwas, pval > 0)

commonsnps <- qtl$SNP[qtl$SNP %in% gwas$SNP]

qtl <- qtl[qtl$SNP %in% commonsnps, ] %>% dplyr::arrange(SNP)

gwas <- gwas[gwas$SNP %in% commonsnps, ] %>% dplyr::arrange(SNP)

input <- merge(qtl, gwas, by="SNP", all=FALSE, suffixes=c("_1","_2"), sort=F)

type1 <- "quant"

if(!all(is.na(qtl$type))){

type1 <- qtl$type[1]

}

if(!is.null(qtl_type)){

type1 <- qtl_type

}

type2 <- "cc"

if(!all(is.na(gwas$type))){

type2 <- gwas$type[1]

}

if(!is.null(gwas_type)){

type2 <- gwas_type

}

if(is.null(qtl_S)){

qtl_S <- qtl$s

}

if(is.null(gwas_S)){

gwas_S <- gwas$s

}

if("N" %in% colnames(qtl) && !all(is.na(qtl$N))){

qtl_samplesize <- qtl$N[1]

}

if(type1 == "cc"){

dataset1 <- list(pvalues=input$pval_1,

type=type1, s=qtl_S[1],

N=qtl_samplesize, MAF=input$maf_1,

snp=input$SNP)

}else{

dataset1 <- list(pvalues=input$pval_1,

type=type1, N=qtl_samplesize, MAF=input$maf_1, snp=input$SNP)#qtl Y轴

}

if(type2 == "cc"){

dataset2 <- list(pvalues=input$pval_2, type=type2,

s=gwas_S[1], N=gwas_samplesize,MAF=input$maf_2,

snp=input$SNP)

}else{

dataset2 <- list(pvalues=input$pval_2, type=type2, N=gwas_samplesize,

MAF=input$maf_2, snp=input$SNP)

}

if("position" %in% names(dataset1) && "position" %in% names(dataset2)){

dataset2$position <- dataset1$position

}

file_aa = paste0(get_basename(qtl_file), "_", get_basename(gwas_file))

coloc_res <- coloc::coloc.abf(dataset1,

dataset2)

if(is.null(to_dir)){

time_str <- format(Sys.time(), "%Y%m%d%H%M%S")

to_dir <- paste(time_str, "for_coloc_res", sep="_")

}

to_dir<-check_dir(to_dir)

coloc_res_name <- paste0(to_dir, "/coloc_res_", file_aa, ".csv")

a = as.data.frame(coloc_res$summary)

data.table::fwrite(a, coloc_res_name, row.names = T, col.names = T)

coloc_snp_results <- paste0(to_dir, "/coloc_data_", file_aa, ".csv")

data.table::fwrite(coloc_res$results, coloc_snp_results, row.names = T, col.names = T)
