# Supplementary material for: Exploration of potential novel drug targets for rheumatoid arthritis by plasma proteome screening
Source: PLoS Comput Biol. 2025 Sep 25;21(9):e1013333. doi: 10.1371/journal.pcbi.1013333 (PMC12463240; doi:10.1371/journal.pcbi.1013333)
Supplement: S3 Code — (S3_Code.DOCX) [file pcbi.1013333.s023.docx]

**S3 Code. Core code for SMR analysis**

#Use smr software to run smr

cmd_exe = "smr.exe"

bfile = "EUR"

gwas_file = "Outcomes "

beqtl_file = "cis-eQTLs-full_eQTLGen_AF_incl_nr_formatted_20191212.new.txt_besd-dense.epi"

out_file = "res"

thread-num = 24

c <- sprintf("%s --bfile %s --gwas-summary %s --beqtl-summary %s --out %s/smr_whole --thread-num %s",

cmd_exe, bfile, gwas_file, beqtl_file, out_file, thread_num)

system(c)
