# Supplementary material for: Exploration of potential novel drug targets for rheumatoid arthritis by plasma proteome screening
Source: PLoS Comput Biol. 2025 Sep 25;21(9):e1013333. doi: 10.1371/journal.pcbi.1013333 (PMC12463240; doi:10.1371/journal.pcbi.1013333)
Supplement: S4 Code — (S4_Code.DOCX) [file pcbi.1013333.s024.docx]

**S4 Code. Core code for ROC**

library(pROC)

library(ROSE)

library(readr)

expr <- read_csv("expr.csv")

group <- read_csv("rocgroup.csv")

expr_t <- as.data.frame(t(expr[,-1]))

colnames(expr_t) <- expr$...1

expr_t$sample <- colnames(expr)[-1]

merged <- merge(expr_t, group, by = "sample")

merged$group <- factor(merged$group, levels = c("HC", "RA"))

set.seed(123)

train_idx <- sample(nrow(merged), size = 0.7 * nrow(merged))

train <- merged[train_idx, ]

test <- merged[-train_idx, ]

train_bal <- ovun.sample(group ~ ., data = train[, -1], method = "over")$data

genes <- setdiff(colnames(expr_t), "sample")

auc_values <- sapply(genes, function(gene) {

roc_obj <- roc(test$group, as.numeric(test[[gene]]), levels = c("HC", "RA"))

as.numeric(auc(roc_obj))

})

barplot(auc_values, names.arg = genes, las = 2, col = "skyblue",

main = "AUC of each gene on test set", ylab = "AUC")

print(round(auc_values, 3))

genes <- setdiff(colnames(expr_t), "sample")

n <- length(genes)

ncol <- ceiling(sqrt(n))

nrow <- ceiling(n / ncol)

par(mfrow = c(nrow, ncol), mar = c(4, 4, 3, 2))

colors <- rainbow(n)

for (i in seq_along(genes)) {

gene <- genes[i]

roc_obj <- roc(test$group, as.numeric(test[[gene]]), levels = c("HC", "RA"))

auc_val <- round(auc(roc_obj), 3)

plot(roc_obj, main = gene, col = colors[i], lwd = 2,

legacy.axes = TRUE, cex.main = 1.2)

abline(a = 0, b = 1, lty = 2, col = "gray")

text(0.6, 0.2, paste0("AUC: ", auc_val), col = colors[i], cex = 1.3)

}
