# Supplementary material for: Exploration of potential novel drug targets for rheumatoid arthritis by plasma proteome screening
Source: PLoS Comput Biol. 2025 Sep 25;21(9):e1013333. doi: 10.1371/journal.pcbi.1013333 (PMC12463240; doi:10.1371/journal.pcbi.1013333)
Supplement: S5 Code — (S5_Code.DOCX) [file pcbi.1013333.s025.docx]

**S5 Code. Core code for Random Forest Models**

library(randomForest)

library(caret)

library(pROC)

library(ggplot2)

group_df <- read.csv("group.csv", row.names = 1)

expr_df <- read.csv("expr_matrix.csv", row.names = 1)

common_samples <- intersect(rownames(group_df), rownames(expr_df))

group_df <- group_df[common_samples, , drop = FALSE]

expr_df <- expr_df[common_samples, ]

data <- cbind(expr_df, Group = as.factor(group_df$group))

set.seed(2024)

train_index <- createDataPartition(data$Group, p = 0.7, list = FALSE)

train_data <- data[train_index, ]

test_data <- data[-train_index, ]

rf_model <- randomForest(Group ~ ., data = train_data, ntree = 500, importance = TRUE)

print(rf_model)

pred_class <- predict(rf_model, newdata = test_data)

confusion <- confusionMatrix(pred_class, test_data$Group)

print(confusion)

pred_prob <- predict(rf_model, newdata = test_data, type = "prob")[, "RA"]

roc_obj <- roc(test_data$Group, pred_prob, levels = c("HC", "RA"), direction = "<")

plot(roc_obj, col = "darkred", main = "Random Forest ROC Curve", lwd = 2)

auc(roc_obj)

varImpPlot(rf_model, type = 2, main = "Feature Importance (Mean Decrease Gini)")
